# A SARS-CoV-2 variant with the 12-bp deletion at E gene

**DOI:** 10.1080/22221751.2020.1837017

**Published:** 2020-10-29

**Authors:** Yi-Sheng Sun, Fang Xu, Qi An, Chen Chen, Zhang-Nv Yang, Hang-Jing Lu, Jian-Cai Chen, Ping-Ping Yao, Jian-Min Jiang, Han-Ping Zhu

**Affiliations:** a Key Lab of Vaccine, Prevention and Control of Infectious Disease of Zhejiang Province, Zhejiang Provincial Center for Disease Control and Prevention, Hangzhou, People’s Republic of China; b Shanghai King-cell biotechnology Co., Ltd., Shanghai, People’s Republic of China

**Keywords:** COVID-19, SARS-CoV-2, E gene mutant, virus replication, immunogenicity

## Abstract

The coronavirus disease 2019 (COVID-19) pandemic is still ongoing and has become an important public health threat. This disease is caused by a new coronavirus named severe acute respiratory syndrome coronavirus-2 (SARS-CoV-2) infection, and so far, little is known about this virus. In this study, by using plaque purification, we purified two SARS-CoV-2 virus strains from the same specimen, one named F8 containing a 12-bp deletion in the E gene and the other named 8X containing the wild-type E gene. There was no significant difference in the viral titer and infectivity of these two strains. The S protein content of the F8 viral culture was 0.39 μg/ml, much higher than that of 8X. An inactivated vaccine made from the F8 strain could trigger high levels of the IgG titer and neutralizing antibody titer, which could last for at least 6 weeks and were significantly higher than those from the 8X strain at 1 and 3 weeks post vaccination, respectively. In conclusion, we reported that both the E gene mutant and wild-type SARS-CoV-2 strains were isolated from the same clinical sample by plaque purification. A 12-bp deletion in the E gene was important for SARS-CoV-2 replication and immunogenicity.

COVID-19 is a severe emerging respiratory infection disease, and the patients have a wide range of symptoms such as fever, fatigue, pneumonia and acute respiratory failure [1]. Although the transmission route is not quite clear yet, airborne transmission through droplets is the dominant route for the spread of COVID-19 [2]. On 11 March 2020, WHO had declared COVID-19 a pandemic. By 4th October 2020, 34,804,348 confirmed-cases of COVID-19 and 1,030,738 death were reported worldwide [3]. COVID-19 is so destructive that it reminds people of the great “Spanish flu” pandemic of 1918–1919. The pathogen was first isolated and identified as a new type of coronavirus by the China CDC [4]. It was classified into *Betacoronavirus* genus and named as severe acute respiratory syndrome coronavirus 2 (SARS-CoV-2) by the International Committee on Taxonomy of Viruses. SARS-CoV-2 virus shares approximately 79% sequence identity with the SARS virus [5]. However, unlike the SARS virus, SARS-CoV-2 has lower mortality and obviously stronger infection ability than SARS. The COVID-19 pandemic is still ongoing and has become a substantial public health threat.

Coronaviruses harbour the largest known RNA viral genomes in nature. The S, N, M, and E proteins are the four major structural proteins of coronaviruses, while the E protein is the smallest one. The E protein is an integral membrane protein located at the viral envelope and the E protein monomer can oligomerize to form an ion channel protein known as a viroporin [6]. The E protein has three domains: a short hydrophilic amino (N)-terminal domain, a long carboxy (C)-terminal domain, and a transmembrane domain (TMD). The TMD is important for the E protein homotypic interactions, and mutations at the TMD, such as N15A and V25F, could inhibit oligomerization of the E protein into a pentameric ion conductive pore [6]. The E protein plays important roles in viral morphogenesis, replication, and pathogenesis [7]. A SARS virus lacking the E gene would have a lower viral titer, and this variant strain might be used for the development of attenuated SARS vaccines [8]. We previously reported a SARS-CoV-2 virus strain with a 12-bp deletion in the E gene [9]. To date, no other studies about E gene deletion or mutations of SARS-CoV-2 have been reported.

In this paper, we isolated two SARS-CoV-2 strains with both wild-type and mutant E genes from the same sample using plaque purification. We compared the viral titer, infectivity, S protein content, and immunogenicity of both strains. Our results indicated that a 12-bp deletion in the E gene was important for SARS-CoV-2 replication and immunogenicity.

## Methods

### Ethics statements

All the experiments related to human specimens were approved by the Ethics Committee of the Zhejiang Provincial Center for Disease Control and Prevention (ZJCDC) in China. All the experiments related to live SARS-CoV-2 viruses were carried out in a biosafety level 3 (BSL-3) laboratory in ZJCDC.

### Virus and cells

The SARS-CoV-2 clinical strains F8 and 8X were purified from the pharyngeal swab of a male COVID-19 patient in Hangzhou. Specimens were treated with 25 μg/ml amphotericin B, 1000 units/ml of penicillin, and 1000 μg/ml of streptomycin for 1 h at 4°C prior to Vero cell infection. When the cytopathogenic effect (CPE) occurred, cell cultures were collected and designated passage 1 virus. The procedure was repeated and passage 2 virus was obtained. The 50% tissue culture infective dose (TCID_50_) was calculated using standard methods as mentioned previously [10]. Vero cells were grown in MEM (Gbico, USA) with 10% fetal bovine serum (Every Green, China) at 37°C in a 5% CO_2_ incubator.

### Plaque purification

Vero cells were grown in 6-well plates at a density of 1*10^6^ cells/well. When a confluent cell monolayer was formed, 10-fold serial dilutions of the passage 2 virus were seeded and incubated for 1 h. Then, the viral culture was discarded and replaced with 0.6% agarose gel in cell culture medium. After incubation for 2 days, a second agarose layer containing 0.1% neutral red was added. One day later, the plaques were picked by sterile pipette tips and used to infect Vero cells. When CPE was observed, the cell culture was collected and RNA was extracted from the cultured plaques using an RNeasy Extraction Kit (Qiagen, Germany) according to the manufacturer’s instructions. The cDNA was synthesized by a PrimeScript™ II 1st Strand cDNA Synthesis Kit (Takara, Japan). PCR was performed using a set of primers (wE-F1/R1, Table 1) specific for the E gene, and the PCR products were sequenced to identify the mutant and wild-type E genes. Other PCR products obtained using the primers wE-F2/R2 were analysed by 3% agarose gel electrophoresis. The SARS-CoV-2 strains containing the mutant and wild-type E genes were named as F8 and 8X, respectively. Vero cells were infected at a multiplicity of infection (MOI) of 0.01. Both purified F8 and 8X strains were passaged for 3 generations and then used in the following experiments.

**Table 1.** Primers and probe for the E gene.

Name	Sequence
mE-F1	5′-ATGTACTCATTCGTTTCGGAAG-3′
mE-R1	5′-AATATTGCAGCAGTACGCACA-3′
mE-probe	5′-FAM-TTCGTGGTAACACTAGCCATCCTT-BHQ-3′
wE-F1	5′-TACACAGTTACTTCACTTCAGAC-3′
wE-R1	5′-AGTACTGTTGTACCTCTAACAC-3′
wE-F2	5′-TCATTCGTTTCGGAAGAGACAG-3′
wE-R2	5′-AATATTGCAGCAGTACGCACAC-3′
mE-F1/R1 and probe were used for RT-PCR; wE-F1/R1 were used for sequencing and wF2/R2 for gel electrophoresis.	

### Vaccine preparation and inactivation validation

Viruses were propagated in Vero cells in 75 cm^2^ flasks and harvested 2–3 days after inoculation. Spike (S) protein quantitation was performed by the SARS-CoV-2 (2019-nCoV) Spike Detection ELISA Kit (Sino Biological, China). β-Propiolactone was used for virus inactivation and aluminum hydroxide was added as the adjuvant at a final concentration of 0.5 mg/ml. Validation of virus inactivation was performed by blind passage in series for three generations. Briefly, 1 ml of inactive SARS-CoV-2 virus or MEM as the negative control was inoculated into Vero cells in 25 cm^2^ flasks, and the cells were cultured at 37°C for 4 days. The cell culture was the first passage. Then, 1 ml of the supernatant of the first passage was inoculated into another Vero cell monolayer in 25 cm^2^ flasks and incubated for 4 days. This was the second passage. The procedure was repeated to prepare the third passage. No CPE was observed in any of the three generations.

### Mouse experiments

Six-week old, female BALB/c mice were randomly divided into 5 groups, and each group contained six mice. The S protein content was used to quantify the vaccine dose and mice were immunized intraperitoneally with two doses (135 ng or 27 ng/dose). Immunization procedure was performed at day 0 and boosted at day 7. The control group was immunized with aluminum hydroxide at a concentration of 0.5 mg/ml in phosphate-buffered saline (PBS).

### Enzyme-linked immunosorbent assay (ELISA)

ELISA plates (Corning, USA) were coated with 50 ng of RBD protein per well (Genscript, China) at 4°C overnight, and blocked with 10% FBS in PBS containing 0.5% Tween-20 for 1 h at 37°C. Serum samples were diluted and applied to each well for 1 h at 37°C. Plates were incubated with rabbit anti-mouse IgG-HRP antibody (Abcam, UK) and developed with the substrate 3,3′,5,5′-tetramethylbenzidine (TMB). The absorbance was detected at 450 nm, and an OD_450_ value greater than 2.1-fold of the background value was regarded as positive.

### Neutralization assay

The plaque reduction neutralization test (PRNT) was performed using the 12# SARS-CoV-2 virus strain [9]. Serum samples from immunized mice were inactivated at 56°C for 0.5 h and diluted serially. The 2-fold-diluted sera were mixed and incubated with an equal volume of viral culture (100 TCID_50_) at 37°C for 1 h. The mixture was added onto Vero E6 cells in 6-well plates and incubated at 37°C for another 1 h. Then, the viral culture was discarded and replaced with 0.6% agarose gel in viral culture medium. After incubation for 2 days, a second agarose layer containing 0.1% neutral red was added. The plaque numbers were counted one day later and serum dilutions leading to 50% plaque reductions (PRNT_50_) were calculated as titers.

#### RT-PCR

RNA was extracted from 14 clinical samples and viral cultures as mentioned in the Plaque purification section. The TaqMan method was applied to detect the mutant E gene in an ABI 7500 PCR machine using the One Step PrimeScript™ RT-PCR Kit (Takara, Japan). Primers and probes are shown in Table 1. The RT–PCR conditions were as follows: reverse transcription reaction: 42°C for 10 min, 95°C for 10 sec; PCR: 95°C for 5 sec, 60°C for 34 sec, repeated for 40 cycles. Quantitation of the E gene mutant in the original specimen (P0), and passage 1 and 2 (P1 and P2) of viral culture was performed by TaqMan RT–PCR. Serial 10-fold dilution of the virus stock (strain F8 at 10^6.5^ TCID_50_/ml) was prepared, and the standard curve for the E gene mutant was developed by Tagman RT–PCR using the primer and probe designed specifically for the E gene (Table 1). Viral loads of the E gene mutant at passage 1 and 2 of viral culture were calculated according to the standard curve.

### Immunofluorescence assay

Briefly, Vero cells were seeded onto 35 mm dishes and infected with the F8 or 8X virus at an MOI of 0.01. Two days later, the cells were fixed with 80% ice-cold acetone for 20 min, permeabilized with 0.2% Triton X-100 for 5 min and blocked with 10% goat serum for 1 h. Cells were incubated with the recovered COVID-19 patient serum at a dilution of 1:200 at 4°C overnight. Then, the cells were stained with FITC-labeled anti-human IgG antibody (Sigma-Aldrich, USA) for 1 h at room temperature. DAPI was applied to stain the nuclei for 5 min. The fluorescence was observed using fluorescence microscopy (Leica, Germany) and images were processed by Photoshop (Adobe, USA).

### Statistical analysis

All data were analysed with GraphPad Prism 8.0 or Microsoft Excel 2007 software. The Student’s t-test was performed with *p* < 0.05 between two groups considered as statistically significant.

## Results

### A 12-bp deletion of the E gene found in the SARS-CoV-2 virus strain

**Figure 1.**
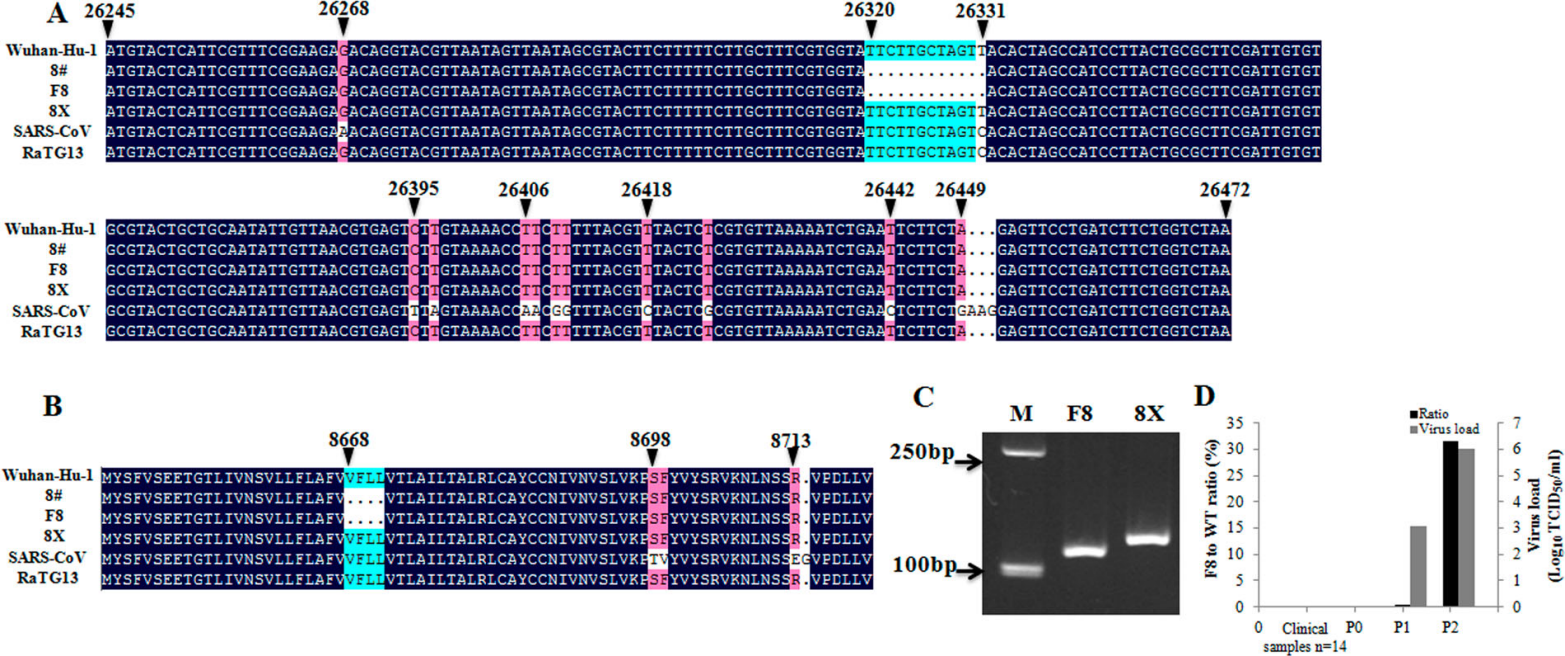
A 12-bp deletion in the E gene found in SARS-CoV-2. Nucleotide (A) and amino acid (B) sequence alignments of the E genes of SARS-CoV Tor2, bat coronavirus RaTG13, and four SARS-CoV-2 strains (8#, F8, 8X, Wuhan-Hu-1). (C) Electrophoresis of the partial E gene PCR products from F8 and 8X. M: marker. (D) Detection of the E gene mutant in 14 clinical specimens, and in the original specimen and passages 1 and 2 (P0, P1 and P2) of viral culture before plaque purification. Viral loads of P1 and P2 were quantitated by Tagman RT-PCR based on the standard curves obtained from 10-fold serial dilutions of the F8 strain with 10^6.5^ TCID_50_/ml.

We isolated two strains of SARS-CoV-2 virus named F8 and 8X from the swab sample of a COVID-19 patient by plaque purification. Both strains were sequenced and a 12-bp deletion in the E gene at the position of 26320–26331 was found in the F8 strain, while no deletion in the E gene was detected in the 8X strain. We performed a sequence alignment of these sequences with the E gene sequences from Wuhan-Hu-1, SARS-CoV Tor2, bat coronavirus RaTG13 strains, and a SARS-CoV-2 strain (8#) isolated from the same patient without plaque purification previously [9] (Figure 1(A)). Apart from the deletion section (26320–26331), other nucleotide sequences were the same among the four SARS-CoV-2 virus strains. The T at the position of 26331 in SARS-CoV-2 was changed to C in the SARS-CoV Tor2 and bat coronavirus RaTG13 strains. We also did an amino acid sequence alignment (Figure 1(B)), and a “VFLL” amino acid deletion was found correspondingly in the F8 and 8# strains, while the other three strains, except SARS-CoV Tor2, had the same amino acid sequences. Gel electrophoresis showed that the PCR product of E gene from the F8 strain was shorter than that from the 8X strain (Figure 1(C)). These results suggested that two SARS-CoV-2 strains containing either the wild-type or mutant E gene were purified from the same specimen.

### Detection of the mutant E gene in clinical samples

To explore whether other clinical samples contained a similar SARS-CoV-2 strain lacking a 12-bp sequence in the E gene, we used RT–PCR to detect the mutant E gene specifically. As shown in Figure 1(D), no signal was detected in 14 clinical samples. Passage 0 of viral culture was the original specimen of F8 and 8X, and passages 1 and 2 were viral cultures before plaque purification. No signal was detected at passage 0, similar to other clinical samples. The proportion of the mutant E gene strain increased sharply from 0.5% in passage 1 to approximately 31.6% in passage 2, with viral loads of 10^3.1^ and 10^6.0^ TCID_50_/ml, respectively. These results indicated that the SARS-CoV-2 E gene mutant was rare in clinical samples.

### Viral titer, replication, and infectivity of the F8 and 8X strains

**Figure 2.**
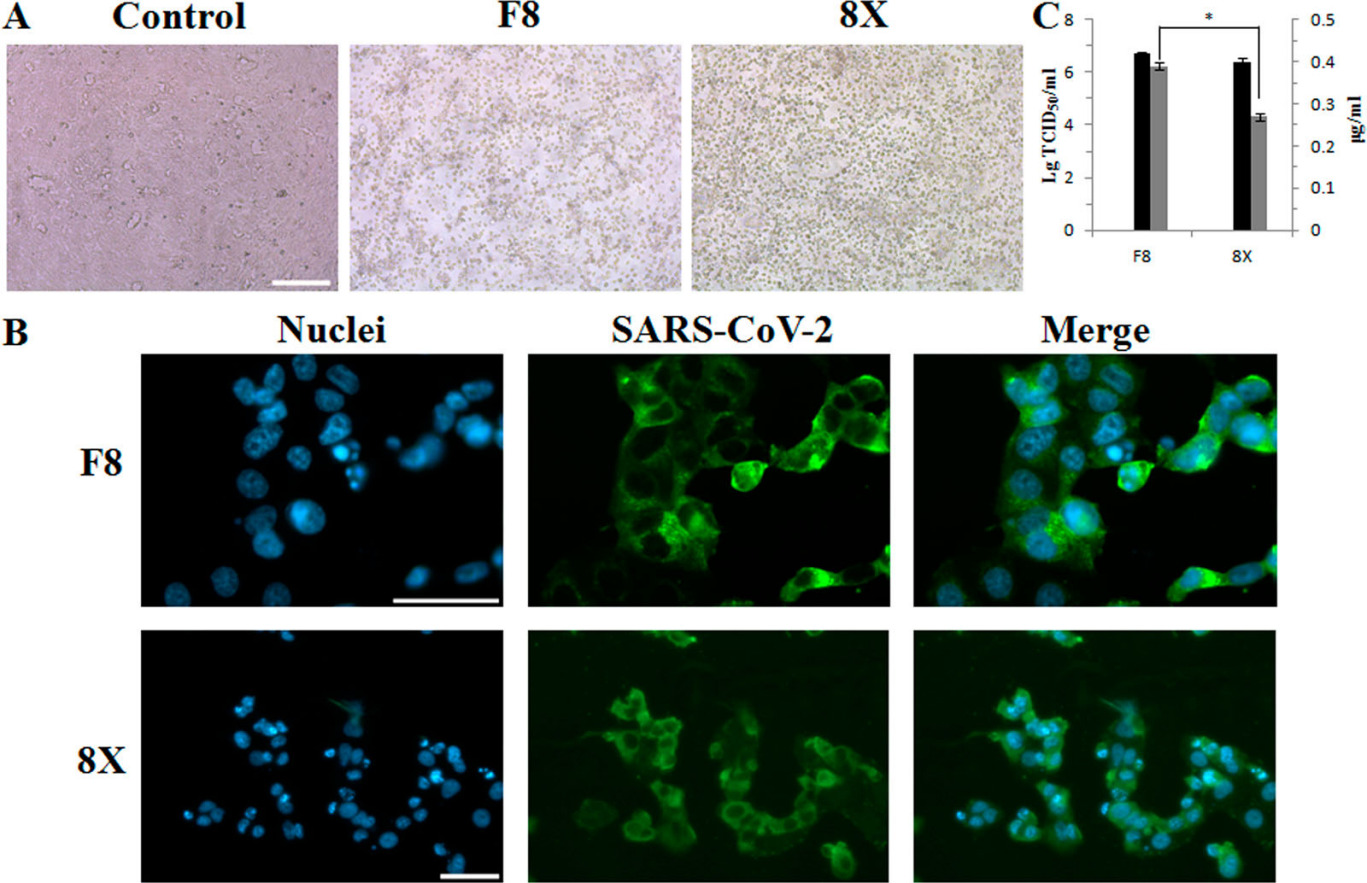
Viral titer, S protein content, and infectivity of the F8 and 8X strains. (A) Vero cells were infected with the F8 or 8X virus at an MOI of 0.01 for 48 h. PBS was used as the negative control. Bar: 200 μm. (B) Vero cells were infected with the F8 or 8X virus for 48 h and an indirect immunofluorescence assay using the positive serum from a COVID-19 recovered patient was performed. SARS-CoV-2 virus was stained with the FITC-labeled secondary antibody (Green). Nuclei were stained with DAPI (Blue). Bar: 100 μm. (C) Viral titers (Black) and S protein content (Grey) of the F8 and 8X strains. *Significant difference between the two groups (*p* < 0.05).

We propagated the F8 and 8X strains in Vero cells. After infection for 48 h, a typical cytopathic effect (CPE) was observed in Vero cells. As shown in Figure 2(A), adherent epithelial cells became round and many of them floated in the medium. Indirect immunofluorescence method was used to detect the viral infection. Both the F8 and 8X strains can infect and enter into Vero cells successfully (Figure 2(B)). SARS-CoV-2 virions were located and replicated in the cytoplasm, not in the nuclei. We also measured the viral titer and the S protein content of the two strains. The viral titer of the F8 strain virus was approximately 1*10^6.5^ TCID_50_/ml, while that of the 8X strain was 1*10^6.2^ TCID_50_/ml, showing no significant difference (Figure 2(C)). However, there was a significant difference in the S protein content. The S protein content of the F8 viral culture was 0.39 μg/ml, while it was 0.27 μg/ml for 8X. Our results indicated that both the F8 and 8X strains can infect Vero cells, however, the S protein content of the F8 viral culture was higher than that of 8X.

### Immunogenicity of F8 and 8X in mice

**Figure 3.**
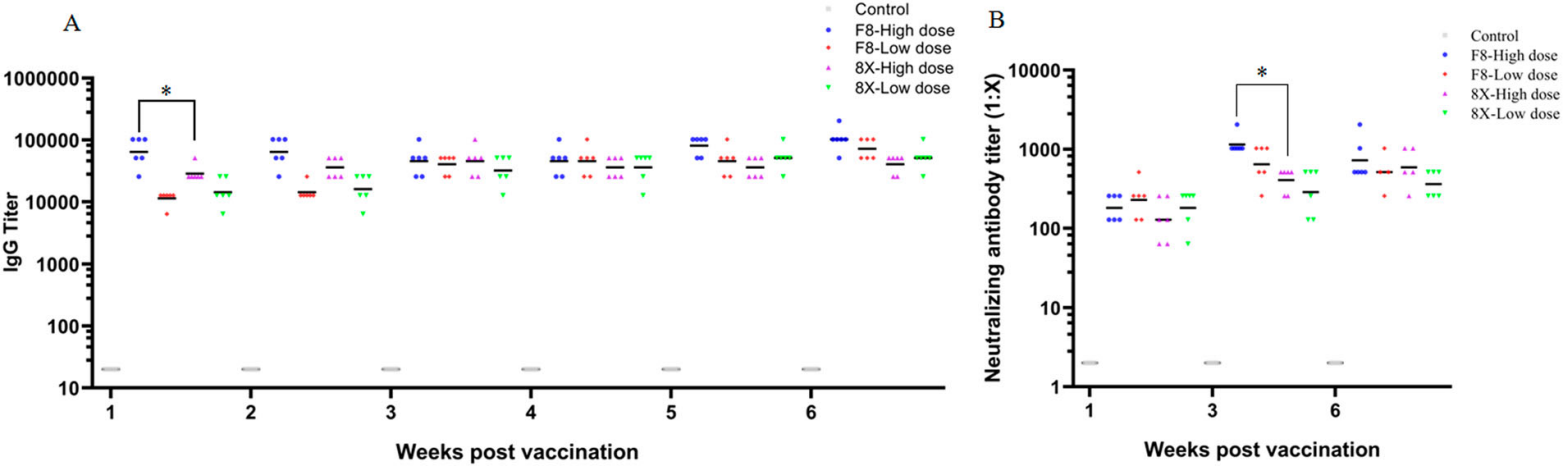
Immunogenicity of the F8 and 8X vaccines in mice. Mice were immunized with F8 and 8X vaccines at day 0 and booster at day 7. Aluminum hydroxide at a concentration of 0.5 mg/ml in PBS was used as the negative control. The S protein content was used to quantify the vaccine dose. High dose: 135 ng of S protein. Low dose: 27 ng of S protein. Each group had six mice. Serum was collected at 1, 2, 3, 4, 5, and 6 weeks post booster vaccination. (A) SARS-CoV-2-specific IgG antibody titers were determined by ELISA. *Significant difference between the two groups (*p* < 0.05). (B) Serum samples at 1, 3, and 6 weeks post the booster vaccination were obtained, and the neutralizing antibody titer was measured by the plaque reduction neutralization test. Serum dilutions leading to 50% plaque reductions (PRNT_50_) were calculated as titers. *Significant difference between the two groups (*p* < 0.05).

To determine the immunogenicity of the F8 and 8X strains, we used β-propiolactone to prepare the inactivated SARS-CoV-2 vaccines from F8 and 8X viral cultures. S protein quantitation was used to quantify the vaccine dose. The high dose of F8 and 8X vaccines were 135 ng of S protein each, with a low dose of 27 ng each. As shown in Figure 3(A), we found that both F8 and 8X vaccine-immunized mice could develop high SARS-CoV-2-specific IgG titers. One week after booster vaccination, the geometric mean titer (GMT) of IgG was 64507 in the F8 high dose group, much higher than 28735 in the 8X high dose group. However, in both F8 and 8X low dose groups, the GMTs of IgG were 11403 and 14367 respectively, with no significant difference (Figure 3(A)). We detected the IgG titers every week after vaccination for 6 weeks, and the IgG titers in all F8 and 8X groups remained at a high level at 2–6 weeks post vaccination. The GMTs of neutralizing antibody (NAb) were 181 and 228 in the high and low dose of F8 groups at the first week post vaccination (Figure 3(B)). Then the GMTs increased to 1149 and 406 at three weeks post vaccination, significantly higher than the value of 645 and 287 observed in the 8X group. At six weeks post vaccination, the NAb titers of both the F8 and 8X groups remained at a high level, similar to the IgG titers, and there was no significant difference between the F8 and 8X groups. Our results demonstrated that both F8 and 8X vaccines could induce high SARS-CoV-2-specific IgG titers and neutralizing antibody titers in mice. The F8 vaccine triggered a higher level of IgG titer and NAb titer than the 8X vaccine at 1 and 3 weeks after vaccination, respectively.

## Discussion

SARS-CoV-2 virus is an RNA virus that has a high rate of mutation [11]. Evidence showed that a single amino acid change in the spike protein (D614G) would lead to a 2.6- to 9.3-fold increase in the infectious titer. Currently, the SARS-CoV-2 variant with the G614 form of the spike protein is the dominant pandemic strain in the world [12]. It is critical to study the SARS-CoV-2 variants as well as their infectivity, pathogenicity, and immunogenicity. We previously reported a SARS-CoV-2 variant with a 12-bp deletion in the E gene [9]. Interestingly, by using plaque purification, we isolated two SARS-CoV-2 strains from the same patient’s specimen: the F8 strain containing the mutant E gene and the 8X strain containing the wild type E gene. Both the F8 and 8X strains could infect Vero cells, and there was no significant difference between the viral titers of the two strains. The F8 viral culture contained significantly more S protein than the 8X viral culture, indicating more SARS-CoV-2 virions and higher viral replication in the F8 viral culture. However, due to the facility limitations, we cannot currently perform animal challenge experiments in our laboratory, and the pathogenicity of the two strains has not been determined yet. Special attention should be paid to the pathogenicity of the E gene variant in the future.

E gene is a small but important structural protein for coronaviruses (CoVs). It is involved in many viral cycle processes, such as assembly, budding, and envelope formation. The E gene is conserved in coronaviruses [8]. However, according to the GISAID database (as of 25th May 2020), more than 40 amino acid mutations of the E gene were found from 4085 SARS-CoV-2 genomes. The E gene of SARS-CoV-2 seemed to have a high mutation rate. However, no mutations were reported in the 26320–26331 region. Our group was the first to report this in-frame deletion in the E gene, and compare the infectivity and immunogenicity between the strains with the mutant and wild-type E gene. Although E gene deletion in SARS virus could lead to a significantly reduced viral titer, we did not find any difference in viral titer of the SARS-CoV-2 E gene variant. Using ELISA method, higher S protein content was measured in the F8 viral culture than in the 8X culture, indicating more virions in the E gene mutant viral culture. According to the nucleotide sequence and the corresponding domain in the SARS-CoV virus, the 12-bp deletion sequence (26320–26331) in the F8 strain was located at the TMD of E gene, which is responsible for E protein homo-oligomeric multimer formation and influences the ion channel activity of the E protein as a viroporin. The ion channel activity of E viroporin is important for viral replication and pathogenesis. Mutations at the TMD residues V25F and N15A could block the ion channel activity of the CoV E viroporin, while mutations close to V25F such as F26L or L27S could compensate for the loss of ion channel activity [6]. The E gene mutant strain F8 had an amino acid deletion “VFLL” at the positions 25–28. However, in our study, the E gene mutant strain F8 seemed to produce more SARS-CoV-2 virions than that of wild type 8X. Based on the previous studies on the SARS virus, E gene deletion would not stop virus production but rather would cripple virus production [6]. Cells infected with recombinant SARS-CoV-ΔE (rSARS-CoV-ΔE) would have a higher proportion of immature virions [13]. Though the F8 viral culture seemed to have more SARS-CoV-2 virions than 8X, we believe that a much higher number of SARS-CoV-2 virions in the F8 viral culture were immature, similar to the recombinant SARS-CoV-ΔE. Immature virions may not induce the CPE in Vero cells. This might explain why the F8 viral culture contained more SARS-CoV-2 virions than the 8X culture but with no difference in viral titer. We also found some SARS-CoV-2 virus detection methods and virus quantification methods based on the E gene of SARS-CoV-2 virus [14,15]. Fortunately, the locations of the RT–PCR primers and probes were not in the deletion domain. In the future, we hope that the 12-bp deletion in the E gene will be considered before detection method development.

It is critical to know whether the E gene variant F8 was already existed in patients or was generated during viral culture and propagation. Through limited clinical specimen detection, we did not find any variants with a 12-bp deletion in the E gene. We also did not detect any E gene mutants in the original specimen (P0) of the F8 strain. In the passages 1 and 2 of the F8 strain before plaque purification, the ratio of the E gene mutant strain to the wild type increased sharply from 0.5% to approximately 31.6%. Our RT–PCR detection method may not be sensitive enough. The variant F8 with the 12-bp deletion in the E gene was more likely generated as a result of viral adaption to Vero cells. We also found that the viral titer of the F8 strain was a litter higher than that of the 8X strain, and the F8 strain exhibited CPE slightly earlier than the 8X strain (data not shown), although there are no significant difference in these data. Viral adaption to the Vero cells might explain why the E gene mutant F8 can grow slightly better, and produce more S protein, than the wild-type 8X in Vero cells.

Immunogenicity is critical for vaccine development. High levels of neutralizing antibody titers were found in rSARSCoV-E-immunized hamsters and rSARS-CoV-MA15-E immunized BALB/c mice. All the immunized animals could be protected from SARS-CoV virus challenge [16,17]. In this study, we used β-propiolactone to prepare the inactivated SARS-CoV-2 vaccines from F8 and 8X viral cultures, and BALB/c mice were immunized with the same dose of antigen quantified by the S protein. The seroconversion was 100% and IgG titers were over 10, 000 in all F8 and 8X groups at the first week post vaccination. However, the GMT of SARS-CoV-2-specific IgG in the F8 high dose group was significantly higher than that in the 8X high dose group at the first week after vaccination. We also found that the GMT of the neutralizing antibody increased from 181 to 1149 at three weeks post vaccination in the F8 high dose group, while in the 8X high dose group, the GMT of the neutralizing antibody was 406, which was significantly lower than that in the F8 group. The neutralizing antibody can bind viruses and block viral entry into cells, thus protecting the host. The NAb titer level is a key index of the humoral immunity. It seemed that an inactivated vaccine prepared from the E gene mutant viral culture triggered a quicker humoral immune response than that from the wild type viral culture.

In conclusion, we report an E gene mutant and a wild type SARS-CoV-2 strain isolated from the same clinical sample by plaque purification. The E gene mutant strain F8 exhibited higher S protein content and triggered a quicker humoral immune response than the E gene wild-type strain 8X, indicating that the 12-bp deletion in the E gene was important for SARS-CoV-2 replication and immunogenicity. Since SARS-CoV-2 virus is a newly emerging virus, further research is required, especially regarding the pathogenicity of F8.
